# Molecular and Microenvironmental Determinants of Glioma Stem-Like Cell Survival and Invasion

**DOI:** 10.3389/fonc.2017.00120

**Published:** 2017-06-16

**Authors:** Alison Roos, Zonghui Ding, Joseph C. Loftus, Nhan L. Tran

**Affiliations:** ^1^Departments of Cancer Biology and Neurosurgery, Mayo Clinic Arizona, Scottsdale, AZ, United States; ^2^Department of Biochemistry and Molecular Biology, Mayo Clinic Arizona, Scottsdale, AZ, United States

**Keywords:** glioblastoma, invasion, glioma stem-like cells, survival, stem-cell niches

## Abstract

Glioblastoma multiforme (GBM) is the most frequent primary brain tumor in adults with a 5-year survival rate of 5% despite intensive research efforts. The poor prognosis is due, in part, to aggressive invasion into the surrounding brain parenchyma. Invasion is a complex process mediated by cell-intrinsic pathways, extrinsic microenvironmental cues, and biophysical cues from the peritumoral stromal matrix. Recent data have attributed GBM invasion to the glioma stem-like cell (GSC) subpopulation. GSCs are slowly dividing, highly invasive, therapy resistant, and are considered to give rise to tumor recurrence. GSCs are localized in a heterogeneous cellular niche, and cross talk between stromal cells and GSCs cultivates a fertile environment that promotes GSC invasion. Pro-migratory soluble factors from endothelial cells, astrocytes, macrophages, microglia, and non-stem-like tumor cells can stimulate peritumoral invasion of GSCs. Therefore, therapeutic efforts designed to target the invasive GSCs may enhance patient survival. In this review, we summarize the current understanding of extrinsic pathways and major stromal and immune players facilitating GSC maintenance and survival.

## Introduction

Glioblastoma multiforme (GBM) is the most frequently occurring primary brain tumor in adults (1). GBMs are differentiated from lower grade tumors by the presence of atypical nuclei, high mitotic activity, and areas of necrosis and microvascular hyperplasia (2). The standard of care for primary GBM includes maximal safe surgical resection and a combination of radiation therapy and temozolomide (TMZ) chemotherapy (3). The prognosis and survival of patients with GBM remains dismal with a median survival time of approximately 15 months from the time of diagnosis and a 5-year survival rate of 5% (4, 5). The poor prognosis is due, in part, to GBM cells invading into the surrounding brain parenchyma and inter and intra-tumoral heterogeneity, which confer resistance to traditional and contemporary treatment modalities (6–8).

Recent analyses have been focused on the identification of genetic alterations in the patient’s tumor as a potential means for individualized therapy. Commonly, this approach utilizes biopsy samples from the tumor core, which is subsequently removed during surgical resection (8–10). Unfortunately, this approach provides little insight into the genetic alterations in the residual invasive cells that give rise to tumor recurrence or the microenvironmental factors that influence their survival. The process of invasion is complex and highly orchestrated. An invasive cell must detach from the primary tumor, adhere to extracellular matrix (ECM) components, secrete enzymes that degrade the surrounding matrix, undergo morphological changes to facilitate locomotion, and migrate through surrounding microenvironmental structures (11). To effectively improve patient survival, there is a significant need to enhance our understanding of the molecular mechanisms and microenvironmental factors that drive cell invasion and improve targeting of this population to thwart tumor recurrence.

Currently, it is assumed that recurrence results from glioma stem-like cells (GSCs), since isolated GSCs are inherently chemo- and radioresistant, express enhanced levels of neuronal stem cell markers and are highly invasive (12–17). GSCs are a small subpopulation of tumor cells that self-renew and proliferate to maintain tumor growth (18). GSCs are enriched in distinct niches in vascular and hypoxic regions and are isolated utilizing cell surface markers including CD133, CD44, and CD15 (19). Intracranial injection of CD133^+^ cells produces highly invasive tumors that recapitulate the heterogeneity of the parental tumor (20, 21). Additionally, CD133^+^ cells are enriched after radiotherapy, and CD133 expression is associated with poor patient prognosis in GBM (14, 22). Similarly, CD44 functions as a cell surface marker for several cancer stem cells and has been used to enrich for GSCs (23, 24).

Studies into genomic factors that regulate GBM invasion have compared the gene expression profiles of cells in the invasive rim to cells in the bulk tumor core both *in vitro* and in clinical specimens. Results have revealed differential activation of transcription factors and significant gene expression differences in antiapoptotic and survival pathways in the invasive cells relative to cells in the tumor core (25–27). However, the invasive cells do not manifest recurrence alone but recruit and exploit microenvironmental cells to sustain and promote survival and invasion. This review summarizes the paracrine, autocrine, and intrinsic molecular pathways that have been reported to facilitate GSC maintenance and survival.

## GBM Perivascular Niche Supports GSC Maintenance

Glioblastoma multiforme is a vascularized tumor characterized by aggregates of proliferating endothelial cells (ECs) referred to as microvascular hyperplasia. Angiogenesis is vital for supporting and maintaining rapid tumor growth (28). GSCs migrate toward and are enriched in the abnormal tumor vascular niche and immunohistochemical staining of GBM tumors shows CD31^+^ ECs surrounded by CD133^+^ GSCs (29–31). GSCs promote tumorigenesis by secreting vascular endothelial growth factor (VEGF) that induces EC migration and subsequent angiogenesis (29). The importance of angiogenesis in the function of GSCs is demonstrated by preclinical studies with the neutralizing VEGF antibody bevacizumab, which depletes the tumor vasculature and specifically inhibits tumor growth of GSC-derived xenografts *in vivo* (29, 32).

GSC secretion of VEGF is induced by the CXCL12/CXCR4 ligand/receptor pair (33, 34). In response to CXCL12, CD133^+^ GSCs upregulate VEGF production in a PI3K/Akt-dependent manner (33). Treatment with a CXCR4 antagonist or with the PI3K inhibitor, LY294002, can reduce VEGF production and inhibit growth and angiogenesis of tumor xenografts formed by GSCs (33). Furthermore, inhibiting CXCR4 signaling suppressed the invasive phenotype of GSCs and sensitizes these cells to radiation (35).

Endothelial cells secrete several factors that confer pro-survival and invasive properties to GSCs (29). For instance, the angiopoietin (Ang1)-Tie2 receptor interaction plays a critical role in the invasive phenotype of GSCs. In response to EC-derived Ang1, the tyrosine kinase Tie2 receptor is activated on GSCs and promotes the expression of adhesion proteins, including N-cadherin and integrin β1, to facilitate GSC invasion (36). In fact, integrin β1 has been reported to be critical for diffuse infiltration in GBM (37). In addition, sonic hedgehog (Shh) secreted by CD31^+^ ECs within the perivascular niche can promote sustained GBM tumor growth *in vivo* and self-renewal of GSCs by activating Patched1 and GLI signaling (38, 39). Inhibition of Shh-GLI signaling reduces GSC self-renewal and *in vivo* tumorigenesis suggesting that Shh, in part, is important for GSC survival. Additionally, paracrine factors secreted from ECs in the vascular niche activate the mTOR pathway and promote expansion of GSCs (40).

GSCs can physically interact with vascular cells in the niche, and this interaction initiates, supports, and maintains tumor growth and promotes angiogenesis (29). L1CAM, a neural adhesion molecule that regulates neural growth and migration during development, is overexpressed in GBM and is required for GSC survival and proliferation (41). GSCs directly interact with ECs, induce EC migration, and promote angiogenesis *via* an L1CAM-integrin αvβ3 mechanism (42). Knockdown of L1CAM results in downregulation of Olig2, a critical transcription factor for proliferation and maintenance of GSCs (41, 43, 44). In addition, activated Notch signaling promotes self-renewal and the invasive GSC phenotype (45). Immunofluorescence staining of primary GBMs demonstrates that GSCs expressing high levels of the Notch1 and Notch2 receptors are localized adjacent to Notch-expressing ECs (46). ECs express the Notch ligands DLL4 and JAGGED1 that activate Notch receptors on the surface of GSCs through cell-to-cell contact and promote GSC self-renewal (46). EC-secreted nitric oxide also activates Notch signaling that results in GSC self-renewal and glioma initiation (47). Inhibition of Notch signaling with a γ-secretase inhibitor results in decreased GSC self-renewal, depletes ECs in the vascular niche, and promotes GSC sensitivity to radiation therapy (48, 49).

## Hypoxia Induces GSC Survival

Hypoxia and multifocal necrosis are hallmark features of GBM tumors and arise through the unregulated proliferation of tumor cells without sufficient supporting vasculature (50). Hypoxia plays a critical role in tumor progression, metabolism, metastasis, invasion, and therapeutic resistance (51, 52). Necrotic areas are surrounded by hypercellular regions termed pseudopalisades, which are microscopic structures unique to GBM. Pseudopalisades express higher levels of stem cell markers and are hypothesized to be waves of cells migrating away from hypoxic areas (53, 54). In GBM, GSCs have been reported to be enriched in hypoxic regions that promote maintenance and induces the expansion of GSCs (55, 56). Studies have shown that the hypoxic niche promotes the reprogramming of glioma non-stem cells into a cancer stem cell-like phenotype and regulates GSC self-renewal (30, 55, 57). Cellular responses to hypoxia are largely regulated by a family of transcription factors known as hypoxia-inducible factors (HIFs) (58). The expression of HIF2α increases with hypoxia, correlates with CD133 expression, and enhances the self-renewal capacity of GSCs (56, 59). HIF2α mediates the hypoxia-induced expression of stem cell markers specifically in GSCs, including Sox2, Oct4, and c-Myc (30, 57).

Hypoxia also induces the expression of HIF1α, and ablation of HIF1α expression decreases the CD133^+^ population and inhibits the invasive capability of glioma cells (56, 60). HIF1α is required for hypoxia-mediated maintenance of GSCs *via* activation of Notch pathway. Ablation of HIF1α or inactivation of the Notch pathway inhibits the hypoxia-mediated maintenance of GSCs (61). Furthermore, HIF1α can interact with and stabilize the intracellular domain of Notch, thus activating the Notch signaling pathway (61). In fact, hypoxia-dependent activation of Notch results in a decrease in E-cadherin expression and subsequent cell invasion (62). Treatment with a Notch inhibitor blocks hypoxia-induced invasion and promotes GSC apoptosis (62, 63).

## Acidosis Regulates GSC Invasion and Stemness

The pH of GBM tumors is much lower compared to normal tissue (64). Low pH derives from enhanced metabolism and low oxygenation of GBM tumors. Low pH promotes the mRNA expression of HIF2α and stem cell markers, including Oct4, Olig2, and Nanog (64). Tissue pH also regulates the expression of VEGF in a MAPK-dependent mechanism, which potentiates tumor growth (65, 66). Acidosis and hypoxia cooperate to induce HIF transcription factors and promote GSC maintenance through the stress-induced chaperone protein HSP90 pathway. Furthermore, inhibition of HSP90 by shRNA knockdown or pharmacological inhibitors impairs the self-renewal and tumorigenic capacities of GSCs induced by acidosis (67).

The sodium–hydrogen exchanger isoform 1 (NHE1) protein is one factor that mediates the cellular response to pH (68). GBM cells express high levels of NHE1 to maintain homeostatic intracellular pH levels. NHE1 potentiates GBM cell migration by altering matrix metalloproteinase (MMP) activity and through direct interaction with the ERM complex protein ezrin (69).

## Tumor-Associated Macrophages/Microglia (TAMs) Support GSC Maintenance

The central nervous system has resident immune cells, or microglia, that are responsible for eliciting an immune response (70, 71). Immunohistochemical staining of GBM tumors has shown high levels of infiltrating microglia, which constitute around 40% of brain tumor mass (72, 73). Upon recruitment to the tumor microenvironment, microglia become immunosuppressive and promote angiogenesis and invasion (74, 75). Ablation of microglia with clodronate liposomes or minocycline inhibits the invasion of GBM cells, supporting an important role for microglia in GBM invasion (75–77). GSCs play a vital role in the recruitment of microglia and macrophages and also secrete factors that promote pro-tumorigenic functions of microglia (78). GSCs upregulate interleukin 6 (IL6) release from microglia *via* toll-like receptor 4 (TLR4) signaling, which enhances invasion and glioma growth (79). Glioma cells also produce macrophage colony-stimulating factor, which induces microglia secretion of insulin-like growth factor binding protein 1 that promotes angiogenesis (80).

The ECM surrounding brain tumors serves as an impediment to invasion. MMPs, including gelatinases and membrane-type MMPs (MT-MMPs), are necessary to digest ECM components and facilitate invasion into the surrounding brain parenchyma (81). GSCs express high levels of the gelatinase MMP2, which is synthesized as a proenzyme and must be activated by cleavage. In a paracrine mechanism, GBM cells activate TLR signaling in microglia, which results in MT1-MMP expression (76, 77). Microglia-derived MT1-MMP activates GSC-derived MMP2 and promotes GBM invasion (82). Microglia also express high levels of osteopontin (OPN), a known ECM component that regulates GBM invasion. OPN interacts with CD44 and induces cleavage of CD44 in proneural GBM. The CD44 intracellular domain promotes GLSC self-renewal and an aggressive tumor phenotype, in part, *via* activation of HIF2α (83).

Microglia also release several growth factors that affect glioma cell invasion and survival. Treatment with microglia-conditioned media activates the protein tyrosine kinase 2 beta (Pyk2) signaling pathway in glioma cells (84). Pyk2 promotes cell invasion by activating MAP4K4 signaling in glioma cells (85, 86). In addition, microglia produce the epidermal growth factor (EGF) and enhance EGF-signaling pathways in glioma cells. Pharmacological inhibition of EGFR prevents microglial-induced invasion in glioma cells (87). The transforming growth factor-beta (TGFβ) is another factor that is secreted by microglia. TGFβ pathway is dysregulated in glioma and plays a key role in invasion *via* upregulation of MMPs and αvβ3 integrin expression (88–90). Microglia secrete TGFβ1 that activates the TGFβR2 pathway in GSCs and results in increased MMP9 expression and subsequent invasion (91, 92). TGFβ inhibitors block GSC-dependent tumor initiation (23).

In addition to microglia, tumor-associated macrophages are enriched in GSC niches (93, 94). These peripheral monocyte-derived macrophages are recruited by GSCs to the microenvironment *via* a periostin/integrin αvβ3-dependent mechanism (95). Upon recruitment to the GSC niche, TAMs adopt a pro-tumorigenic M2 phenotype and secrete IL6 and IL10 to promote the proliferation of GSCs in hypoxic niches (96).

## Astrocytes Support GSC Maintenance

GSCs synergize with resident brain cells to promote tumorigenesis. There are reports that astrocytes promote the invasive capacity of GSCs, in part, by direct cell contact and secreting proteins associated with cell invasion (97, 98). Astrocytes secrete chemokines and cytokines, including IL6 and TGFβ2, that promote GSC invasion (97). Astrocyte-derived IL6 decreases the radiosensitivity of GSCs *in vitro* in a Stat3-dependent mechanism (99). Treatment with WP1066, a JAK/STAT3 inhibitor, enhances radiosensitivity of GSCs in a xenograft model (99). GFAP-positive astrocytes also secrete Shh, which activates GLI signaling and GSC stemness (38). Cross talk between astrocytes and glioma cells results in the activation of astrocyte-derived MMP2 and subsequent glioma migration. In a pathway mediated by the urokinase-type plasminogen activator-plasmin cascade, glioma-derived plasmin activates astrocyte-secreted MMP2. MMP2 degrades ECM components and facilitates glioma cell dispersal (98).

## ECM Factors Mediate GSC Invasion

GSCs interact with the ECM during invasion. Although the composition of the brain tumor ECM is still being elucidated, there are high levels of hyaluronic acid (HA) surrounding invasive GBM tumors compared to normal brain tissue (100). HA is a glycosaminoglycan that provides structural support and regulates cell adhesion and migration (101). HA interacts with specific cognate receptors including CD44 and receptor for hyaluronate-mediated motility (RHAMM) (102). High levels of CD44 and RHAMM correlate with poor patient prognosis in GBM (102). HA interaction with CD44 or RHAMM activates downstream signaling cascades in GSCs that result in self-renewal, multidrug resistance, and cell invasion (102–106). Blocking the glioma cell-HA interaction inhibits GBM invasion and anchorage-independent growth (107). In addition, brevican, an extracellular hyaluronan-binding protein specific to the brain that is overexpressed in GBM, is enriched in the GSC niche (108, 109). Cleavage of brevican enhances the invasion of glioma cells (110). In contrast, brevican knockdown inhibits the tumorigenicity of GBM cells suggesting that brevican may play an important role in glioma progression (108, 109).

GSCs can secrete MMPs to remodel the surrounding ECM and create space for migration. GSCs express high levels TLR2, which upon activation enhances GSC invasion *via* upregulation of MMP2 and MMP9 (111). GSCs also express high levels of MMP13, which specifically enhances migration and invasion (112).

ADAM proteins are characterized by their metalloproteinase activity and integrin-receptor binding and have been implicated in several important cellular processes, including cell migration (113). ADAM17 has been shown to promote self-renewal capability of GSCs and inhibit GSC differentiation *via* Notch signaling (114). ADAM17 also promotes the invasion of CD133^+^ GSCs isolated from human glioblastoma cell line U87 through the EGFR/PI3K/AKT signaling pathway (115). ADAM9 has been found to mediate tenascin-C induced invasiveness of GSCs, and inhibition of ADAM9 attenuates GSC invasion (116).

## Conclusion

Until the recent survival benefits afforded by the introduction of Novocure Optune, the survival of patients with primary GBM has remained unchanged since the Stupp protocol was implemented in 2005 (3, 117). Recent clinical trials, including altering TMZ doses or targeting specific genomic alterations, have failed to improve patient overall survival (118–120). This suggests that a paradigm shift for treating this devastating disease is warranted.

**Figure 1:**
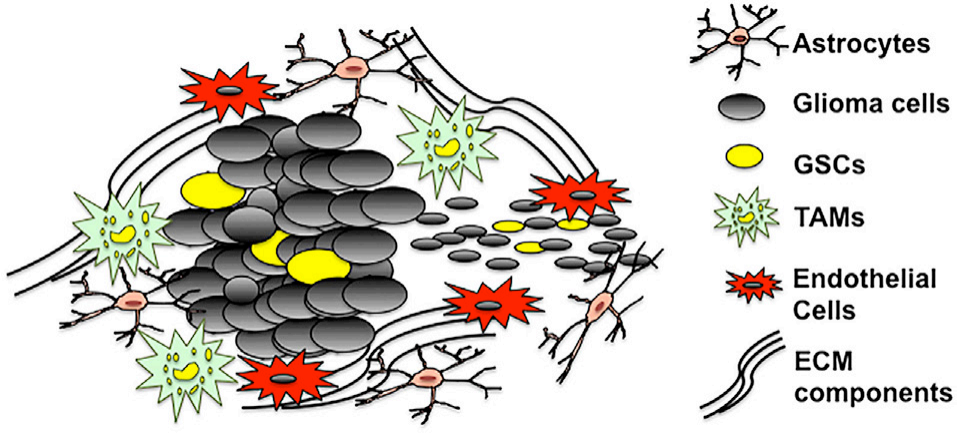
The GSC microenvironment. GSCs are enriched in both hypoxic and vascular niches surrounded by extracellular matrix (ECM) components and cells of different lineages including astrocytes, non-stem glioma cells, tumor-associated microglia/macrophages, and endothelial cells. The tumor microenvironment provides a fertile environment that supports and propagates GSCs.

Glioblastoma multiforme tumors are cellularly heterogeneous and consist of GSCs, non-GSCs, microglia/TAMs, astrocytes, and neurons (Figure 1). GBM tumors appear to have a cellular hierarchy with GSCs at the apex. The GSC niches are symbiotic environments, with GSCs and non-tumoral cells reciprocally promoting tumorigenesis.

**Figure 2:**
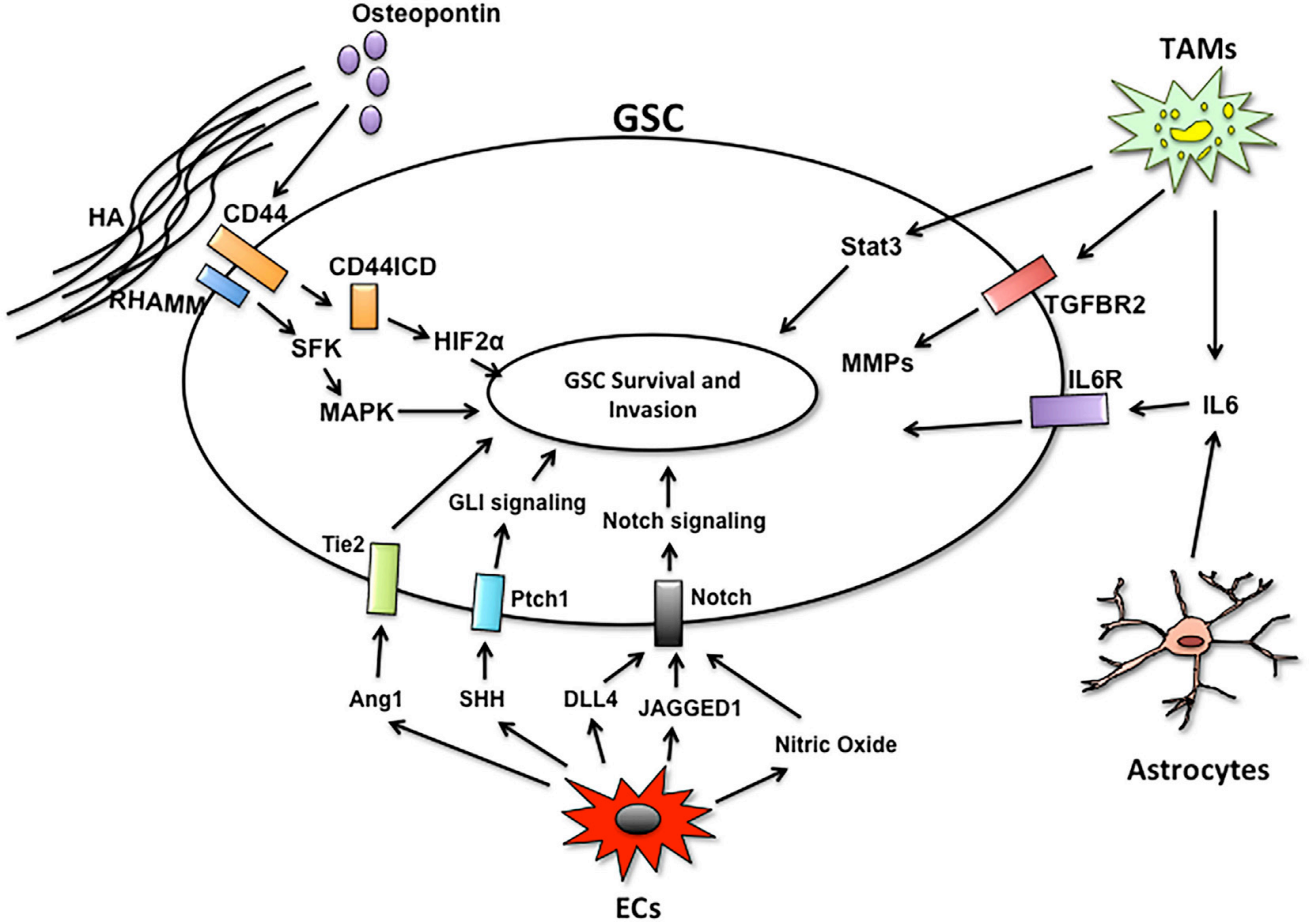
Microenvironmental cells and paracrine pathways facilitating GSC invasion. The GSC niches are comprised of endothelial cells (ECs), tumor-associated macrophages/microglia (TAMs), astrocytes, and extracellular matrix components. Non-tumor cells within the niche support GSC survival and invasion by secreting factors that activate key signaling pathways in GSCs to maintain self-renewal and survival.

Currently, it is thought that tumor recurrence derives from GSCs. GSCs are highly invasive and resistant to the current modes of therapy. This subpopulation is maintained and supported through direct interactions and paracrine signaling from microenvironmental cells (Figure 2). Microglia, the brain resident immune cells, cooperate with GSCs to promote invasion and tumor progression. In addition to secreting factors that potentiate GSC invasion and self-renewal, glioma cells and microglia share pro-tumorigenic pathways that may serve as nodes of intervention. Microglia express high levels of the TNFR receptor TROY, which activates microglial migration in a PYK2-dependent manner (121). Targeting microglia migration toward the GSC niche with propentofylline, a small molecule inhibitor of TROY signaling, mitigates the pro-tumorigenic functions of microglia and decreases tumor growth (122). Moreover, TROY is overexpressed in glioma cells and inhibiting TROY signaling blocks invasion (123, 124). Thus, targeting TROY could serve a dual function of inhibiting paracrine and autocrine regulators of glioma invasion.

GSCs are enriched in microvascular niches, which support GSC survival and invasion. Although preclinical models have demonstrated survival benefits of antiangiogenic therapies, clinical trials with early administration of bevacizumab failed to improve overall survival in patients with newly diagnosed GBM (125). Additionally, a phase II clinical trial with Tivozanib monotherapy demonstrated Tivozanib could alter the tumor vasculature but did not have antitumoral effect in patients with recurrent GBM (126). Therefore, it is vital that future studies consider not only resistance mechanisms to antiangiogenic therapy but also the symbiosis between GSCs and the microenvironment as a whole and assess the efficacy of antiangiogenic therapies in conjunction with other modes of therapy.

Although recent therapeutic approaches have improved GBM survival, patients still succumb to this devastating disease (117). Our understanding of the impact of the Optune system on GSCs has yet to be determined. If Optune was to be considered as part of standard of care, studies into the effect of tumor treating fields on GSCs and tumor microenvironmental cells are warranted. Additionally, deducing paracrine-regulated pathways mediating GSC survival is critical for mitigating tumor recurrence. Further investigations into the relationships between GSCs and their niches are imperative. A better understanding of the interaction of GSCs and niches will provide new insights to develop new therapeutic strategies for GBM patients. Targeting glioblastoma stem-cell niches may represent a novel strategy for treating GBM.

## Author Contributions

AR wrote most of the manuscript and created the figures. ZD contributed to the text and figures. JL and NT edited the manuscript.

## Conflict of Interest Statement

The authors declare that the research was conducted in the absence of any commercial or financial relationships that could be construed as a potential conflict of interest.
